# Respiratory Sinus Arrhythmia Activity Predicts Internalizing and Externalizing Behaviors in Non-referred Boys

**DOI:** 10.3389/fpsyg.2017.01496

**Published:** 2017-09-11

**Authors:** Wei Zhang, Shawn E. Fagan, Yu Gao

**Affiliations:** ^1^Department of Psychology, Queens College, City University of New York, New York City NY, United States; ^2^Department of Psychology, The Graduate Center, City University of New York, New York City NY, United States; ^3^Department of Psychology, The Graduate Center and Brooklyn College, City University of New York, New York City NY, United States

**Keywords:** respiratory sinus arrhythmia, emotion regulation, externalizing behavior, internalizing behavior, biosocial interaction, gender differences

## Abstract

Atypical respiratory sinus arrhythmia (RSA), a biomarker of emotion dysregulation, is associated with both externalizing and internalizing behaviors. In addition, social adversity and gender may moderate this association. In this study, we investigated if RSA (both resting RSA and RSA reactivity in an emotion regulation task) predicts externalizing and/or internalizing behaviors and the extent to which social adversity moderates this relationship. Two hundred and fifty-three children (at Time 1, mean age = 9.05, *SD* = 0.60, 48% boys) and their caregivers from the community participated in this study. Resting RSA and RSA reactivity were assessed, and caregivers reported children’s externalizing and internalizing behaviors at both Time 1 and Time 2 (1 year later). We found that lower resting RSA (but not RSA reactivity) at Time 1 was associated with increased externalizing and internalizing behaviors at Time 2 in boys, even after controlling for the effects of Time 1 behavioral problems and Time 2 age. Moreover, there was a significant interaction effect between Time 1 resting RSA and social adversity such that lower resting RSA predicted higher externalizing and internalizing behaviors in boys only under conditions of high social adversity. Follow-up analyses revealed that these predictive effects were stronger for externalizing behavior than for internalizing behavior. No significant effects were found for girls. Our findings provide further evidence that low resting RSA may be a transdiagnostic biomarker of emotion dysregulation and a predisposing risk factor for both types of behavior problems, in particular for boys who grow up in adverse environments. We conclude that biosocial interaction effects and gender differences should be considered when examining the etiological mechanisms of child psychopathology.

## Introduction

Externalizing and internalizing behaviors in children are significant risk factors for later mental health problems. The development and progression of these behaviors are heavily influenced by social, environmental, and biological factors. A better understanding of the elements that contribute to such behaviors in youth is critical given the economic and social costs to families and society at large. Increasingly, researchers are examining the contribution of both biological and psychosocial variables to better characterize the development of these externalizing and internalizing behaviors.

Both internalizing and externalizing behaviors are characterized by emotion dysregulation. Emotion regulation commonly operates in a series of steps: monitor and attend to a specific situation, evaluate or reappraise mentally, and update or modify a response in order to accomplish a specific goal (Gross, 2015). According to this model, emotion dysregulation is “a pattern of emotional experience and/or expression that interferes with appropriate goal directed behavior” (Beauchaine, 2012) by disrupting any one of the above steps. In this sense, some individuals with poor emotion regulation have difficulties in controlling emotional responses or inhibiting their behaviors, resulting in externalizing outbursts (Eisenberg et al., 2001). In the context of internalizing behaviors, emotion dysregulation occurs at the level of attention and mental reappraisal, with these individuals unable to inhibit negative thoughts resulting in excessive rumination and sadness (Eisenberg et al., 2001).

One of the accepted objective indicators of emotion regulation is respiratory sinus arrhythmia [RSA; (Beauchaine, 2015)]. RSA measures cardiac activity of the parasympathetic nervous system (PNS) and reflects the variations in inter-beat intervals of the heart rate across respiration (Porges, 1995, 2007). The parasympathetic branch of the autonomic nervous system (ANS) is critically involved in bodily rest and digest and overall homeostasis. Specifically, the PNS slows the heart rate through the activity of the vagus nerve. Increased parasympathetic activity at rest (e.g., high resting RSA) reflects the individual’s readiness to react efficiently to environmental stressors (Gyurak and Ayduk, 2008; Scarpa et al., 2010). Cortical structures, specifically regions within the central neural network (including the orbitofrontal and ventromedial prefrontal cortices, the amygdala, and the periaqueductal gray), feed forward information to preganglionic and parasympathetic nerve cells that in turn innervate the vagus nerve (Thayer et al., 2009). Neural activity in these regions affects downstream parasympathetic activity (Thayer and Lane, 2000; Lane et al., 2009; Thayer et al., 2009; Buchanan et al., 2010). Moreover, emotion dysregulation has been linked to deficiency in the prefrontal brain regions (Beauchaine, 2015). Taken together, RSA, a measure of parasympathetic functioning, is heavily influenced by prefrontal cortex activity and constitutes a good indicator of emotion regulation.

Given that a potential shared mechanism of internalizing and externalizing behaviors is emotion dysregulation, we would expect both behavioral problems to be associated with abnormal resting RSA. Many empirical studies have supported this proposition by showing that either externalizing, internalizing, or both are associated with low resting RSA (Forbes et al., 2006; Dietrich et al., 2007; Hinnant and El-Sheikh, 2009). For example, in their longitudinal study with children, Hinnant and El-Sheikh (2009) showed that lower resting RSA (in conjunction with either RSA suppression or RSA augmentation) predicted increased internalizing symptoms and externalizing symptoms 2 years later. This study highlights not only the importance of resting RSA as a biomarker for emotion (dys)regulation, but also shows the longitudinal predictive power of this biomarker. On the other hand, Dietrich et al. (2007) found externalizing behavior to be associated with elevated resting RSA while internalizing behavior was associated with lower resting RSA; however, the authors report that this physiological pattern may underlie externalizing behaviors that necessitate *increased* emotion regulation, such as proactive aggression versus reaction aggression.

Changes in RSA from baseline to task have also been used to assess parasympathetic reactivity in response to external threats. RSA reactivity can indicate either vagal/RSA withdrawal or vagal/RSA augmentation. During vagal withdrawal, the vagus nerve withdraws its control over the heart, resulting in increased PNS (RSA) reactivity. Appropriate levels of vagal withdrawal are adaptive and necessary for effectively reacting to external threats. In contrast, during vagal augmentation, the vagus nerve increases its control over the heart, resulting in decreased PNS (RSA) reactivity. In certain circumstances vagal augmentation is beneficial, particularly when completing cognitive tasks that require heightened attention and emotional control (DiPietro et al., 1992); however, in most cases vagal augmentation may lead to “increased control of emotionality” that can “attenuate painful experiences and insensitivity to socializing punishments” (Scarpa and Raine, 2004).

Findings linking RSA reactivity and externalizing and internalizing behaviors are mixed. Many studies previously found externalizing and internalizing to relate to blunted and increased RSA reactivity, respectively (Boyce et al., 2001; Calkins et al., 2007; Hinnant and El-Sheikh, 2009; Fortunato et al., 2013). For example, Hinnant and El-Sheikh (2009) found that children with high internalizing symptoms and low resting RSA had excessive RSA withdrawal during emotion tasks, indicating hyper-reactivity. They also saw that those high in externalizing symptoms and that had low resting RSA showed reduced RSA withdrawal, suggesting maladaptive emotional withdrawal during stressful situations (Hinnant and El-Sheikh, 2009). Similarly, children at risk for mixed internalizing and externalizing problems demonstrated consistently elevated levels of RSA withdrawal during various tasks while children at risk for high externalizing problems only displayed the smallest amount of vagal withdrawal compared to the mixed problem and low risk group (Calkins et al., 2007). Additionally, Fortunato et al. (2013) found that children with high RSA withdrawal in response to a negative emotion induction showed increased levels of internalizing, while children with reduced RSA withdrawal to a positive emotion induction showed increased levels of externalizing behavior. Taken together, these findings suggest that children with externalizing behavior alone exhibit diminished PNS reactivity, whereas children with internalizing (on its own or comorbid with externalizing) exhibit excessive PNS reactivity (Boyce et al., 2001; Calkins et al., 2007; Hinnant and El-Sheikh, 2009; Fortunato et al., 2013). However, another study reported that increased RSA withdrawal in children during independent free play was associated with externalizing behaviors (Hastings et al., 2008) and many studies have failed to establish a link between PNS reactivity and externalizing/internalizing problems all together (Gordis et al., 2010).

The absence of a consistent relationship between RSA and externalizing/internalizing problems potentially reflects a failure to consider the impact of environmental influences. Prior research has found that both psychosocial adversity and psychophysiological factors are important for understanding the etiologies of child behavioral problems. Some have demonstrated that psychophysiological abnormalities such as lower resting RSA and abnormal RSA reactivity increase susceptibility to psychosocial risk factors for behavioral deficits. For example, lower levels of resting RSA increased risks for externalizing problems whereas elevated levels of resting RSA buffered against externalizing problems for boys exposed to familial stressors such as inter-parental arguments (El-Sheikh et al., 2001). Similarly, a study on stress exposure and its effect on externalizing/internalizing behaviors showed that childhood adversity (including traumatic events, community violence exposure, and child abuse) and resting RSA interact such that at low levels of resting RSA and increased adversity, depression/withdrawal symptoms increase, but not for adolescents with high resting RSA (McLaughlin et al., 2015). With respect to RSA reactivity, children with high RSA reactivity to a cognitive task demonstrated increased externalizing symptoms under conditions of high marital conflict, and reduced externalizing symptoms under conditions of low marital conflict; externalizing symptoms of children with low RSA reactivity were unaffected by marital conflict (Obradović et al., 2011). Taken together, the combination of low resting RSA or high RSA reactivity and high psychosocial adversity seem to increase risks for both internalizing and externalizing behaviors.

Additionally, evidence of gender differences with respect to psychosocial characteristics and physiological activity is becoming more apparent in the current literature. In one study, childhood stress exposure (i.e., abuse, community violence, trauma, etc.) interacted with resting RSA to predict anxiety/depression problems in male adolescents, but not in female adolescents (McLaughlin et al., 2015). Similarly, young boys at risk for externalizing showed lower resting RSA than low-risk boys but no such difference was observed between high- and low-risk girls (Calkins and Dedmon, 2000). Taken together, evidence suggests that RSA may be a stronger predictor of behavioral problems for males than for females, supporting the proposition that there are distinct mechanisms underlying the development of behavioral problems for the two gender groups (Beauchaine et al., 2008).

Prior studies have principally examined the concurrent associations between RSA and behavioral problems (Calkins and Dedmon, 2000; Calkins et al., 2007; Dietrich et al., 2007; Gordis et al., 2010; Fortunato et al., 2013; McLaughlin et al., 2015). There have been fewer longitudinal studies evaluating RSA and its interaction with environmental factors in predicting behavioral development over time (El-Sheikh and Whitson, 2006; Hinnant and El-Sheikh, 2009; El-Sheikh and Hinnant, 2011; Eisenberg et al., 2012). For example, a study on the longitudinal relationship between RSA, marital conflicts and child behaviors showed that lower initial resting RSA predicted externalizing behaviors from 8 to 11 years in boys (El-Sheikh and Hinnant, 2011). The aforementioned study of Hinnant and El-Sheikh (2009) found that the interaction between resting RSA and RSA reactivity predicted child externalizing/internalizing behaviors 2 years after. Developmental models might further clarify the stability and/or malleability of RSA activity, the process by which RSA may result in maladaptive behavioral development, and the potential of implications for preventive interventions.

In summary, atypical RSA, reflecting emotion dysregulation, is suggested to be a predisposing risk factor for both externalizing and internalizing disorders. In addition, the RSA-behavior problem association may be moderated by psychosocial factors and gender. The current study was conducted in order to examine the developmental trajectory of behavior problems in early childhood through the pre-teen years. Specifically, we aim to examine if RSA offers longitudinal prediction of externalizing or internalizing behaviors in male and female children from the community, and if social adversity moderates this relationship. We targeted children aged 7- to 10-years old in attempt to identify risk factors associated with the emergence and development of conduct disorder that is diagnosed at approximately age 10 [*Diagnostic and statistical manual of mental disorders (DSM-5^®^*); American Psychiatric Association, 2013]. This age range is particularly relevant because previous studies have identified middle childhood as a period during which children are exposed to school-related demands and pathologies involving emotion dysregulation are increasingly diagnosed (Musser et al., 2011). Social adversity was assessed in Time 1 (T1) and psychophysiological measures and behavioral problems were assessed in both Time 1 (T1) and Time 2 (T2) with approximately 1 year apart. We hypothesized that (1) low resting RSA at T1 would predict high internalizing and externalizing behaviors at T2, (2) excessive RSA reactivity at T1 would predict high internalizing behavior at T2 whereas reduced RSA reactivity at T1 would predict high externalizing behavior at T2, and (3) RSA would interact with social adversity such that RSA abnormality in conjunction with high social adversity (but not low adversity) would predict greater externalizing or internalizing behaviors. Consistent with prior literature (El-Sheikh and Whitson, 2006; El-Sheikh et al., 2007; Hinnant and El-Sheikh, 2009), T1 behavioral measures were controlled for in our analyses to examine the influence of T1 RSA and social adversity over and beyond the effects of T1 behavioral measures on T2 measures. Since behavioral problems and pathologies involving emotion dysregulation emerge and increasingly develop during this age range, the effects of age were additionally controlled for in our analyses. In light of growing evidence that the mechanism underlying behavioral problems differs between males and females, we conducted analyses for boys and girls separately, though no formal gender-specific predictions were made given inconsistent findings in the literature.

## Materials and Methods

### Participants

Participants were 253 children (at T1, *M*age = 9.05, *SD* = 0.60, range = 7.51–10.75; at T2, *M*age = 10.05, *SD* = 0.60, range = 8.67–11.75) and their primary caregivers recruited between 2013 and 2014 from an ongoing longitudinal study examining the effects of neurobiological and psychosocial risks on child neurobehavioral development in the metropolitan area of Brooklyn, New York. Families were invited to participate if their children were between 7- and 10-years-old and lived in the study area. Exclusion criteria for participation included children diagnosed with psychiatric disorder, intellectual disability, or a pervasive developmental disorder. A detailed description of the study can be found elsewhere (Gao et al., 2015, 2016; Fagan et al., 2017).

Families who had children that met the eligibility requirements were contacted and 340 families consented to participate for the T1 assessment (48% male; 52% Black, 21% Caucasian, 11% Hispanic, 2% Asian, and 14% mixed/other). Approximately 1 year later at T2, all families were invited for a follow-up assessment, and 253 consented to participate (48% male; 48% Black, 23% Caucasian, 8% Hispanic, 3% Asian, and 18% mixed or other). No significant gender difference was observed in the age of the participants at T1 (*p* = 0.105), however, at T2 boys were slightly older than girls (*t* = 2.38, *p* = 0.018, *d* = 0.30; boys *M* = 10.14, *SD* = 0.57; girls *M* = 9.96, *SD* = 0.61).

Selective attrition analyses via chi-square tests indicated that there were no significant differences between children who did and who did not complete the follow-up assessment according to the child’s gender (*p* = 0.419), ethnicity (*p* = 0.531), levels of externalizing and internalizing behaviors (*p* > 0.332), and RSA measures (*p* > 0.147). However, levels of social adversity were slightly higher among those families that were lost to follow-up (*p* = 0.019).

### Procedure

Children and their primary caregivers were invited to the university for a 2-h lab visit that included psychophysiological testing, a behavioral interview, and neurocognitive and psychosocial assessments at both times. At each visit, participating families received $60 compensation plus transportation reimbursement after the completion of the assessment, and parental consent and child assessment were obtained. The study protocol was approved by the City University of New York Institutional Review Board.

Psychophysiological measures were collected during the psychophysiological testing session, which lasted approximately 40 min and included several tasks. There were two 2-min resting periods, one at the onset and the other at the conclusion of the psychophysiological testing session, during which the children were instructed to sit still and quietly relax. Behavioral interviews and neurocognitive and psychosocial assessments, either before or after the psychophysiological session, were conducted by a trained research assistant.

### Measures

#### Externalizing and Internalizing Behavioral Problems (T1 and T2)

During both T1 and T2 assessments, the accompanying caregiver completed the Child Behavioral Checklist (Achenbach and Rescorla, 2001), a parent-report questionnaire consisting of 112 items. The caregiver rated the child’s behavior within the past 12 months on a three-point Likert scale (0 = *not true*, 1 = *sometimes or somewhat true*, 2 = *very true or often true*). For the purposes of the current study, only the Aggression, Delinquency, Anxious-Depressed, Withdrawal-Depressed, and Somatic-Depressed subscales were used. Reliability tests using Cronbach’s α for T1 and T2 (in parentheses) assessments of Aggression, Delinquency, Anxious-Depressed, Withdrawal-Depressed, and Somatic-Depressed subscales were 0.87(0.85), 0.71(0.65), 0.79(0.74), 0.70(0.72), and 0.80(0.76), respectively. Externalizing behavior score was computed by summing the Aggression and Delinquency subscale scores (at T1, *M* = 6.30, *SD* = 6.08, range = 0–27; at T2, *M* = 5.95, *SD* = 5.83, range = 0–36). Internalizing behavior score was computed by summing the Anxious-Depressed, Withdrawal-Depressed, and Somatic-Depressed subscale scores (at T1, *M* = 6.15, *SD* = 5.95, range = 0–33; at T2, *M* = 5.71, *SD* = 5.84, range = 0–34).

#### Social Adversity

The level of social adversity was assessed via the caregiver reports based on 10 items at T1 (Raine et al., 2002; Gao et al., 2010). A total adversity score was created by adding 1 point for each of the 10 items, which were coded as dichotomous variables (YES “1” or NO “0”), including Divorced Parents (single parent family, remarriage, or living with guardians other than parents), Foster Home, Public Housing, Welfare Food Stamps, Parent Ever Arrested (either parent has been arrested at least once), Parents Physically Ill, Parents Mentally Ill, Crowded Home (five or more family members per room within the home), Teenage Mother (aged 19 years or younger when child was born), and Large Family (having five or more siblings by age 3 years). Higher score indicates higher level of social adversity. In the current sample, the mean was 2.74 (*SD* = 1.88, range = 0–8).

### Psychophysiological Data Recording and Quantification

Electrocardiogram (ECG) and respiration (RSP) data were collected continually at 1,000 Hz using a BIOPAC MP150 system with ECG 100C and RSP100C amplifiers and analyzed oﬄine with the AcqKnowledge 4.2 software (Biopac Systems Inc., Goleta, CA, United States). ECG signal was recorded using ECG100C amplifier with two pre-jelled Ag-AgCl disposable vinyl electrodes placed at a modified Lead II configuration. RSP signal was recorded by putting a respiration belt around the abdomen of the participant at the point of complete exhalation. Saved ECG signals were visually inspected for artifacts, and then converted to interbeat intervals. RSA was derived from the ECG100C amplifier with a band pass filter of 35 and 1.0 Hz and a RSP100C respiration amplifier with a band pass filter of 1.0 and 0.05 Hz. The AcqKnowledge automated function for RSA analysis, which followed the well validated peak-valley method, was utilized to derive RSA (Grossman et al., 1990). RSA was computed in milliseconds as the difference between the maximum and the minimum interbeat intervals during respiration, and higher values indicate greater PNS activity (Gruber et al., 2009). Peak-valley method has been widely used (El-Sheikh et al., 2001; El-Sheikh and Whitson, 2006; Hinnant and El-Sheikh, 2009; Gordis et al., 2010; El-Sheikh and Hinnant, 2011; Viana et al., 2017) and is comparable with other methods of assessing RSA, such as spectral analysis (Grossman et al., 1990).

Resting RSA was computed as the average of the two 2-min resting periods at the beginning and the end of the 40-min psychophysiological testing session. RSA values at the two resting periods were highly correlated (T1, *r* = 0.84, *p* < 0.001; T2, *r* = 0.73, *p* < 0.001). RSA reactivity was computed as the change in RSA from the resting period to the emotion regulation task (Musser et al., 2011). The emotion regulation task (Musser et al., 2011, 2013) consisted of four 2-min film clips taken from the movie *Homeward Bound*, the story of three pets who are left behind when their family goes on vacation and try to find their way home. Children were instructed to either induce or suppress their emotions while psychophysiological activity was recorded. The same sequence of the four movie clips was given to each child: (1) negative induction, (2) negative suppression, (3) positive induction, and (4) positive suppression. A 2-min resting period was placed between the negative and the positive conditions to help restore baseline emotion. The induction condition asked children to facially express the emotion (negative or positive) the main characters were experiencing. In the suppression condition, children were told to think about how the characters were feeling but not to show the emotion (negative or positive) on their faces. RSA values across the four movie conditions were highly correlated (T1, *r* = 0.90-0.92; T2, *r* = 0.80-0.87, *p*s < 0.001), and were therefore averaged to create one RSA measure for the emotion regulation task. Following prior literature (Calkins et al., 2007; Musser et al., 2013), RSA reactivity was computed by subtracting the resting RSA from the average RSA value during the emotion regulation task, with negative values suggesting RSA withdrawal or increased RSA reactivity and positive values reflecting RSA augmentation or reduced RSA reactivity.

### Statistical Analyses

First, sex differences in the study variables were examined using independent samples *t*-tests. Cohen’s *d* was reported for effect sizes of group differences (Cohen, 1988). Then the relationship between RSA measures, externalizing and internalizing behaviors, and social adversity were examined via Pearson correlations for boys and girls separately. Hierarchical multiple regressions were conducted to examine if RSA measures at T1 can predict externalizing and internalizing behaviors at T2, and the potential moderating effect of social adversity. In all regression models, T2 externalizing or internalizing behavior score was the dependent variable, and T1 behavioral problem scores and T2 age were entered as control variables in the first step. In the second step, T1 resting RSA and social adversity were entered to test for their main effects. In the third step, the T1 RSA × social adversity interaction was entered. Significant interaction effects would be probed by conducting the simple slope analysis of T1 RSA on the T2 behavior measure at high (+1 *SD*) and low (-1 *SD*) levels of social adversity values (Aiken et al., 1991). Similar regression analyses were conducted replacing resting RSA with RSA reactivity. All predictor variables were mean centered to create interaction terms and to avoid multicollinearity as well.

### Missing Data, Skewness, and Outliers

All variables (i.e., behavioral measures, RSA, social adversity) were highly skewed (Shapiro–wilk test, *p*s < 0.001), therefore a log transformation was performed for each variable before subsequent analyses were conducted. Among the 253 children, some RSA data (T1, resting RSA, *n* = 36; RSA reactivity, *n* = 104; T2, resting RSA, *n* = 4; RSA reactivity, *n* = 31) were missing due to acquisition or scoring problems, including equipment malfunction, extraneous movement (e.g., too much noise to detect R-waves), and electrode displacement (e.g., improper placement of the respiration belt). Independent samples *t*-tests (or chi-square tests) were performed to examine whether there were differences in gender, race, social adversity and externalizing and internalizing behaviors between participants who were missing resting RSA or RSA reactivity data and the remaining participants. Results indicated that they did not differ on any of the measures (*p*s > 0.312). In addition, a few participants had missing T1 externalizing and internalizing behavior (*n* = 3) and social adversity (*n* = 5) measures. Missing data analysis via the Little’s MCAR test indicated that the data were missing completely at random (Little and Rubin, 1989) (*p* > 0.450). Pairwise deletion was used to maximize all data available on an analysis by analysis basis. Univariate outliers (± 3 *SD*s from the mean) were also examined for each variable. Three outliers were found for T2 RSA measures and removed from all subsequent analyses.

## Results

### Descriptive Statistics, Gender Differences, and Correlations

Compared to girls, boys had higher scores on externalizing behavior at T2 (*t* = 2.08, *p* = 0.038, *d* = 0.26), and marginally higher externalizing scores at T1 (*t* = 1.71, *p* = 0.089, *d* = 0.22). Girls showed more RSA withdrawal compared to boys at T1 (*t* = 2.05, *p* = 0.042, *d* = 0.34). No other gender differences were found (**Table 1**).

**Table 1:** Gender differences in study variables.

	Boys	Girls	Boys			Girls				
Measures	Available	*N*	*Mean*	*SD*	Range	*Mean*	*SD*	Range	*t*	*p*
Externalizing (T1)	120	130	6.86	6.22	0–26	5.78	5.92	0–27	1.71	0.089
Internalizing (T1)	120	130	5.84	6.01	0–33	6.44	5.90	0–28	-0.84	0.404
Externalizing (T2)	121	132	6.65	6.06	0–36	5.30	5.55	0–27	2.08	0.038
Internalizing (T2)	121	132	5.45	5.85	0–34	5.95	5.84	0–32	-0.90	0.368
Social adversity	119	129	2.76	1.86	0–8	2.72	1.90	0–8	0.25	0.803
Resting RSA (T1)	106	111	121.17	61.34	24.75–307.40	128.86	61.00	28.03–319.22	-1.23	0.220
Resting RSA (T2)	119	129	130.02	65.24	39.27–351.30	121.58	56.08	28.91–307.95	0.64	0.521
RSA reactivity (T1)	76	73	1.82	30.54	-88.78–130.51	-8.96	25.53	-77.34–70.59	2.05	0.042
RSA reactivity (T2)	106	114	-4.21	40.59	-118.77–113.63	0.66	31.99	-99.45–115.66	-0.27	0.788

*Independent sample *t*-tests were based on log transformed values. Means and SDs presented were non-transformed values*.

T1 and T2 externalizing and internalizing behaviors were inter-correlated in both boys (*r* ranged from 0.26 to 0.67, *p*s < 0.01) and girls (*r* ranged from 0.52 to 0.73, *p*s < 0.01). T1 resting RSA was negatively correlated with T1 RSA reactivity (boys, *r* = -0.31, *p* = 0.006; girls, *r* = -0.35, *p* = 0.003), and positively correlated with social adversity (boys, *r* = 0.20, *p* = 0.043; girls, *r* = 0.20, *p* = 0.035) and T2 resting RSA (boys, *r* = 0.57, *p* < 0.001; girls, *r* = 0.55, *p* < 0.001). T2 resting RSA was negatively correlated with T2 RSA reactivity (boys, *r* = -0.33, *p* = 0.001; girls, *r* = -0.25, *p* = 0.006). Additionally, in girls social adversity was positively associated with T1 externalizing (*r* = 0.24, *p* = 0.007) and internalizing behaviors (*r* = 0.21, *p* = 0.02) and T2 internalizing behavior (*r* = 0.19, *p* = 0.036). No other significant correlations were found for boys or girls (**Table 2**).

**Table 2:** Correlations by gender.

Measures	1	2	3	4	5	6	7	8	9
1 Externalizing (T1)		0.71**	0.73**	0.53**	0.24**	0.04	0.04	-0.23	-0.08
2 Internalizing (T1)	0.67**		0.52**	0.68**	0.21*	-0.06	0.04	-0.07	0.05
3 Externalizing (T2)	0.55**	0.36**		0.60**	0.16	0.02	0.06	-0.14	-0.13
4 Internalizing (T2)	0.26**	0.48**	0.57**		0.19*	0.00	0.11	-0.03	0.04
5 Social adversity	0.02	-0.06	-0.01	0.01		0.20*	0.10	-0.08	-0.04
6 Resting RSA (T1)	0.05	-0.04	-0.15	-0.16	0.20*		0.55**	-0.35**	0.17
7 Resting RSA (T2)	0.10	0.04	-0.07	-0.15	-0.01	0.57**		-0.09	-0.25**
8 RSA reactivity (T1)	0.09	0.05	0.06	0.14	0.04	-0.31**	-0.05		0.07
9 RSA reactivity (T2)	0.01	0.01	0.02	0.03	-0.03	-0.15	-0.33**	0.22	

*All correlations are based on log transformed data. Area above diagonal shows correlations for girls, area below the diagonal shows correlations for boys. ^∗∗^*p* < 0.01; ^∗^*p* < 0.05*.

### Hierarchical Multiple Regression

Hierarchical multiple regressions were conducted separately for boys and girls to determine whether T1 resting RSA/RSA reactivity, social adversity, and their interaction predicted T2 behavioral problems after controlling for T1 behavioral scores and T2 age. Results are presented in **Tables 3**, **4**.

**Table 3:** Hierarchical regression for T2 externalizing behavior: Predicting effects of RSA, social adversity and RSA × social adversity interaction.

**Externalizing (T2)**								**Externalizing (T2)**							
**Boys**		***b***	***SE***	**β**	***t***	**Δ*R*^2^**	**Δ*F***	**Boys**		***b***	***SE***	**β**	***t***	**Δ*R*^2^**	**Δ*F***
	
Step 1						0.43	37.81^∗∗^	Step 1						0.53	41.28**
	Externalizing (T1)	0.69	0.07	0.70	9.76^∗∗^				Externalizing (T1)	0.71	0.08	0.73	8.88**		
	Age (T2)	0.03	0.05	0.05	0.67				Age (T2)	-0.01	0.05	-0.02	-0.22		
Step 2						0.04	3.90^∗^	Step 2						0.00	0.02
	Rest RSA (T1)	-0.43	0.12	-0.27	-3.55^∗∗^				RSA reactivity (T1)	0.03	0.34	0.01	0.08		
	Social adversity	-0.01	0.11	-0.00	-0.05				Social adversity	-0.02	0.12	-0.01	-0.14		
Step 3						0.04	8.92^∗∗^	Step 3						0.01	0.88
	Resting RSA (T1) × Social adversity	-1.63	0.55	-0.22	-2.99^∗∗^				RSA reactivity (T1) × Social adversity	1.28	1.36	0.08	0.94		
	
**Girls**		***b***	***SE***	**β**	***t***	**Δ*R*^2^**	**Δ*F***	**Girls**		***b***	***SE***	**β**	***t***	**Δ*R*^2^**	**Δ*F***
	
Step 1						0.56	66.27^∗∗^	Step 1						0.58	47.80**
	Externalizing (T1)	0.71	0.07	0.72	10.79^∗∗^				Externalizing (T1)	0.69	0.09	0.71	8.18**	
	Age (T2)	0.10	0.05	0.15	2.25^∗^				Age (T2)	0.13	0.06	0.20	2.32*		
Step 2						0.00	0.01	Step 2						0.00	0.12
	Rest RSA (T1)	-0.03	0.13	-0.02	-0.24				RSA reactivity (T1)	-0.11	0.38	-0.03	-0.30		
	Social adversity	-0.02	0.11	-0.01	-0.16				Social adversity	-0.05	0.13	-0.03	-0.40		
Step 3						0.00	0.59	Step 3						0.00	0.00
	Resting RSA (T1) × Social adversity	-0.39	0.50	-0.05	-0.77				RSA reactivity (T1) × Social adversity	0.02	1.31	0.00	-0.02		

^∗∗^*p < 0.01; ^∗^*p* < 0.05*.

**Table 4:** Hierarchical regression for T2 internalizing behavior: Predicting effects of RSA, social adversity, and RSA × social adversity interaction.

**Internalizing (T2)**								**Internalizing (T2)**							
**Boys**		***b***	***SE***	**β**	***t***	**Δ*R*^2^**	**Δ*F***	**Boys**		***b***	***SE***	**β**	***t***	**Δ*R*^2^**	**Δ*F***
	
Step 1						0.28	19.64^∗∗^	Step 1						0.34	18.23**
	Internalizing (T1)	0.50	0.08	0.53	6.35^∗∗^				Internalizing (T1)	0.52	0.09	0.55	5.58**		
	Age (T2)	0.06	0.05	0.11	1.26				Age (T2)	0.07	0.06	0.11	1.11		
Step 2						0.04	3.00^+^	Step 2						0.02	0.79
	Rest RSA (T1)	-0.36	0.13	-0.24	-2.71^∗∗^				RSA reactivity (T1)	0.44	0.38	0.11	1.16		
	Social adversity	0.14	0.12	0.10	1.14				Social adversity	0.06	0.14	0.04	0.40		
Step 3						0.02	3.31^+^	Step 3						0.00	0.01
	Resting RSA (T1) × Social adversity	-1.08	0.60	-0.16	1.82^+^				RSA reactivity (T1) × Social adversity	-0.17	1.55	-0.01	-0.11		
	
**Girls**		***b***	***SE***	**β**	***t***	**Δ*R*^2^**	**Δ*F***	**Girls**		***b***	***SE***	***β***	***t***	**Δ*R*^2^**	**Δ*F***
	
Step 1						0.50	52.49^∗∗^	Step 1						0.54	41.31**
	Internalizing (T1)	0.65	0.07	0.69	9.57^∗∗^				Internalizing (T1)	0.61	0.08	0.68	7.96**		
	Age (T2)	0.10	0.05	0.16	2.18^∗^				Age (T2)	0.12	0.05	0.21	2.40*		
Step 2						0.00	0.27	Step 2						0.00	0.03
	Rest RSA (T1)	0.09	0.13	0.05	0.66				RSA reactivity (T1)	-0.08	0.33	-0.02	-0.23		
	Social adversity	-0.01	0.11	-0.01	-0.12				Social adversity	-0.01	0.12	-0.01	-0.10		
Step 3						0.00	0.09	Step 3						0.00	0.00
	Resting RSA (T1) × Social adversity	-0.15	0.50	-0.02	-0.31				RSA reactivity (T1) × Social adversity	0.03	1.18	0.00	0.02		

^∗∗^*p < 0.01; ^∗^*p* < 0.05; ^+^*p* < 0.10*.

#### RSA, Social Adversity, and Externalizing Behavior

**FIGURE 1:**
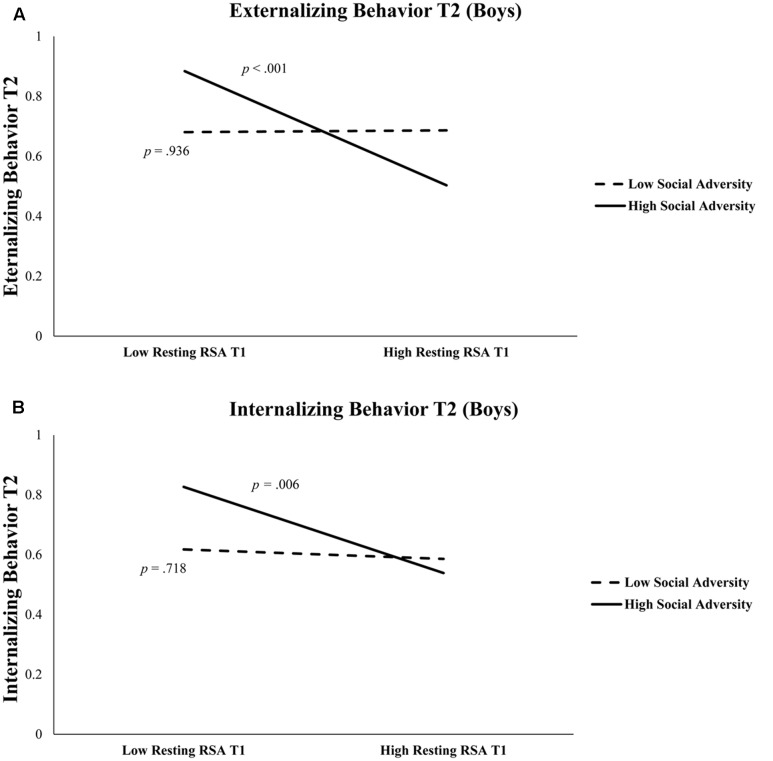
**(A)** Externalizing behavior at Time 2 is predicted by resting RSA × social adversity at Time 1 in boys. High Social Adversity or Resting RSA (+1 *SD*), Low Social Adversity or Resting RSA (–1 *SD*). **(B)** Internalizing behavior at Time 2 is predicted by resting RSA and social adversity at Time 1 in boys. High Social Adversity or Resting RSA (+1 *SD*), Low Social Adversity or Resting RSA (–1 *SD*).

As shown in **Table 3**, in boys, after controlling for T1 externalizing behavior and T2 age, T1 resting RSA significantly predicted T2 externalizing behavior (*b* = -0.43, *t* = -3.55, *p* = 0.001). In addition, the interaction between T1 resting RSA and social adversity was also significant (*b* = -1.63, *t* = -2.99, *p* = 0.004) (**Table 3**). Simple slopes analysis indicated that lower levels of T1 resting RSA were associated with more T2 externalizing behavior in conditions of high social adversity (*b* = -0.81, *t* = -4.09, *p* < 0.001), whereas T1 resting RSA was not linked to T2 externalizing behavior in conditions of low social adversity (*b* = 0.01, *t* = 0.08, *p* = 0.936) (**Figure 1A**). None of the effects were found significant for girls.

Similar regressions with RSA reactivity, social adversity, and their interaction term as independent variables were not significant in predicting T2 externalizing behavior (**Table 3**).

#### RSA, Social Adversity, and Internalizing Behavior

**Table 4** shows regression results predicting internalizing behavior. In boys, after controlling for T1 internalizing behavior and T2 age, lower T1 resting RSA significantly predicted T2 internalizing behavior (*b* = -0.36, *t* = -2.71, *p* = 0.008). The interaction between T1 resting RSA and social adversity was marginally significant for T2 internalizing (*b* = -1.08, *t* = 1.82, *p* = 0.072) (**Table 4**). We further explored this interaction effect and found that lower levels of resting RSA were associated with more internalizing behavior in conditions of high social adversity (*b* = -0.61, *t* = -2.84, *p* = 0.006), although this association was not significant at low levels of social adversity (*b* = -0.07, *t* = -0.36, *p* = 0.718) (**Figure 1B**). None of the models involving RSA reactivity were significant. Finally, none of the models were significant for girls.

#### Follow-up Analyses

As can be seen in **Table 2**, externalizing and internalizing behaviors were highly correlated, consistent with prior literature (Angold et al., 1999). To examine if the above significant main and interaction effects were specific to either externalizing or internalizing behavior, all hierarchical multiple regression analyses were performed again with the T2 non-focal behavior measure added as an additional covariate in the first step. After the T2 internalizing behavior was additionally controlled for while predicting T2 externalizing behavior, lower T1 resting RSA still predicted T2 externalizing behavior in boys (*b* = -0.32, *t* = -2.66, *p* = 0.009). Furthermore, the resting RSA × social adversity interaction effect remained significant (*b* = -1.35, *t* = -2.58, *p* = 0.011), such that lower levels of resting RSA were associated with more externalizing behavior at high levels of social adversity (*b* = -0.63, *t* = -3.28, *p* = 0.001), but not at low levels of social adversity (*b* = 0.05, *t* = 0.30, *p* = 0.764). After the T2 externalizing behavior was adjusted in the model while predicting T2 internalizing behavior, T1 resting RSA predicted T2 internalizing behavior in boys (*b* = -0.26, *t* = -2.01, *p* = 0.047). However, the resting RSA × social adversity interaction became non-significant (*p* = 0.213).

## Discussion

The principle aim of the current study was to investigate if resting RSA or RSA reactivity, an indicator of emotion regulation, interacts with social adversity in prospectively predicting externalizing and internalizing behavior 1 year later in male and female children from the community. We found that in boys, lower resting RSA at T1 was associated with elevated levels of externalizing and internalizing behaviors at T2, over and above the effects of T1 behavioral problems and T2 age. Moreover, significant biosocial interaction effects were observed such that low T1 resting RSA predicted high externalizing and internalizing behaviors in the condition of high but not low social adversity. Follow-up analyses indicated that these predictive effects were stronger for externalizing behavior than for internalizing behavior. In contrast, neither T1 RSA reactivity nor its interaction with social adversity significantly predicted externalizing or internalizing behavior. Finally, none of these variables were significant in predicting behavioral problems in girls.

Consistent with our prediction, low T1 resting RSA was associated with high externalizing and internalizing behaviors T2, although these effects were found for boys only. These results support the theory that low resting RSA is an indicator of poor emotion and attentional regulation due to inefficient prefrontal functioning (Beauchaine, 2001, 2015; Crowell et al., 2006), and demonstrate that resting RSA offers independent longitudinal prediction of behavior problems, over and above the effects of concurrent behavioral problems. Taken together, RSA provides a non-invasive, non-intrusive, and reliable biological tool to assess the PNS activity that reflects emotion regulation, and low resting RSA may serve as a biomarker that specifically predisposes male children to externalizing behavior.

More importantly, we found that the predictive effect of resting RSA was only significant among those with adverse home backgrounds. In fact, low resting RSA and high social adversity in combination predicted the highest levels of both externalizing and internalizing behavioral problems. In contrast, high resting RSA served to protect children from developing either externalizing or internalizing behavioral problems in the face of adverse social environments (**Figure 1**). This is consistent with prior studies that have suggested high resting RSA may buffer against familial and environmental adversity (Eisenberg et al., 2012; McLaughlin et al., 2015), and offer protection under unpredictable or challenging circumstances (Appelhans and Luecken, 2006; Gyurak and Ayduk, 2008). High resting RSA has been associated with adaptive characteristics in children, including better physiological recovery from stress (Lane et al., 1992; Fabes et al., 1993; McLaughlin et al., 2014). Mechanistically, children with higher resting RSA (reflecting increased baseline parasympathetic physiological activity) may be better able to flexibly regulate emotions while experiencing adversity and recover sooner after exposure to high-stress events.

This finding is also in line with the biological sensitivity to context theory that has argued that certain biological phenotypes are differentially affected by environmental stressors. For example, children high in physiological arousal are capable of both flourishing in protective environments and suffering negative behavioral outcomes under hardship (Boyce and Ellis, 2005). One such biological phenotypic marker is low resting RSA, which indicates sympathetic nervous system dominance at rest, resulting in increased vigilance and limited physiological flexibility to meet environmental demands (Thayer and Lane, 2000; Hinnant and El-Sheikh, 2009; Viana et al., 2017). This in turn may predispose individuals to more negative behavioral outcomes under adversity (Boyce and Ellis, 2005). This argument is also partially consistent with the temperament-based theory of antisocial behavior which argues that temperamental constructs such as impulsivity, emotion regulation, and negative affect can contribute to the development of externalizing behaviors (DeLisi and Vaughn, 2014). These issues in contexts of increased adversity are exacerbated, while in positive or nourishing environments may not be as detrimental; alternatively, positive emotionality and good emotion regulation (such as high resting RSA) can protect a child in a highly adverse environment from manifesting externalizing behaviors (DeLisi and Vaughn, 2014).

Interestingly, low resting RSA and its interaction with social adversity were robustly associated with externalizing behavior even after controlling for the effect of concurrent internalizing behavior. However, the effects were somehow attenuated when predicting internalizing behavior after controlling for concurrent externalizing behavior. This finding may indicate some specificity of physiological risk factor, i.e., low resting RSA, for externalizing behavior and further suggest that externalizing and internalizing are two overlapping but distinct constructs, at least in middle childhood. Alternatively, the weaker predictive effects for internalizing behavior may be a function of informant under-reporting of internalizing symptoms that are more difficult to gauge from an outside perspective (Dietrich et al., 2007). Future studies utilizing multi-informant approaches are needed to replicate these findings.

Nonetheless, we found non-significant concurrent relationships between resting RSA and externalizing/internalizing behaviors in both genders at both time points (**Table 2**). While our results replicate a number of null findings of prior research (Hinnant and El-Sheikh, 2009; McLaughlin et al., 2015), they are inconsistent with several previous studies that documented significant associations between resting RSA and behavioral problems at the same time period. For instance, these studies found RSA was associated with concurrent behavior problems (i.e., externalizing or internalizing problems) in 2- to 3-year-old boys with clinical levels of externalizing behaviors (Calkins and Dedmon, 2000), 3- to 9-year-old children born to mothers with childhood-onset depression (Forbes et al., 2006), 8- to 12-year-old externalizing boys with severe conduct problems (Beauchaine et al., 2008), and 10- to 13-year-old externalizers with early-onset behavior problems (at ages 4–5) (Dietrich et al., 2007). Behavior problems assessed in the current community sample may represent a mild form of psychopathology, thus the inconsistency between the present results may be partially attributed to sample differences. Moreover, caution must be taken in interpreting the correlational findings, as we cannot draw any possible conclusion about cause and effect. Using a short-term longitudinal model, we were able to find that children with high resting RSA early in development are less likely to develop behavior problems in the context of high adversity than children with low resting RSA. This pattern further suggests that lower resting RSA represents a diathesis for behavioral problems upon exposure to environmental stressors.

Inconsistent with prior findings, we failed to find significant main or interaction effects involving RSA reactivity in predicting problematic behaviors. Differences in sample characteristics and types of tasks may partly contribute to this inconsistency. For example, significant associations between RSA reactivity and behavioral problems have been reported in a targeted sample of high externalizers from a typical population (Boyce et al., 2001), and preschoolers (*M*age = 5) (Obradović et al., 2011). In addition, these studies have used passive emotional tasks or cognitive tasks (Boyce et al., 2001; Hinnant and El-Sheikh, 2009; Obradović et al., 2011) whereas ours is an effortful task involving explicit emotion regulation. Obradović et al. (2011) argued that elevated RSA activity in a cognitive task could indicate “adaptive engagement” for children in low conflict environments while conferring limitations under cognitive stress in high conflict environments (p. 110). Meanwhile, children with elevated RSA reactivity in an interpersonal task had consistent(ly) higher levels of externalizing behaviors across high and low conflict environments compared to children with low RSA reactivity to the interpersonal task. The mixed emotional and cognitive nature of our task (which is specific to emotion regulation rather than a stress response) makes it difficult to compare with prior studies. Finally, the moderate change of RSA in our sample may indicate that the emotion regulation task may not be strong enough to induce stable RSA reactivity. Future investigations using a consistent category of emotional task and a typical population of children in middle childhood are needed to clarify if RSA reactivity during these tasks can longitudinally predict behavioral problems.

Alternatively, this null finding for RSA reactivity may in part reflect the developmental characteristics of RSA measures in childhood. Prior research has suggested that resting RSA has better longitudinal stability than RSA reactivity (Bornstein and Suess, 2000; Hinnant and El-Sheikh, 2009; Pang and Beauchaine, 2013). For instance, a study in 8- and 12-year-old children with conduct problems and/or depression showed that resting RSA was relatively stable (inter-correlation from 1 year to the next ranged from 0.35 to 0.54), whereas RSA reactivity increased across time (not significantly correlated) (Pang and Beauchaine, 2013). Our sample additionally demonstrated this pattern: while resting RSA values at two times were significantly correlated (*r* ranged from 0.55 to 0.57, *p* < 0.001), RSA reactivity at T1 and T2 were not significantly correlated (*r* ranged from 0.07 to 0.22, *p* > 0.05). However, the degree to which this higher developmental malleability in RSA reactivity (as opposed to baseline measures) influences our findings is unknown.

In both gender groups resting RSA was positively associated with social adversity, supporting the proposition that early-life social adversity may directly alter the HPA axis in order to bring the stress response to more adaptive levels. Early adversity experiences have been related to changes in physiological response to stress, although the impacts on vagal activity have not been clearly demonstrated (Gunnar and Quevedo, 2007). In addition, boys had higher levels of externalizing behavior compared to girls at both time points, whereas girls had higher levels of internalizing behaviors than boys, although the latter differences were non-significant. This is in line with literature showing that the increased levels of internalizing and depressive symptoms in girls emerge during early adolescence (Ge et al., 2001). While it is also possible that boys simply exhibit more externalizing behaviors than girls, a likely alternative is that measures of externalizing behaviors are biased toward the kind of physical aggression more often seen in males (Zoccolillo, 1993; Crick and Grotpeter, 1995). Importantly, the predictive effects of RSA and social adversity were found for boys only. This compliments a growing body of literature showing that boys and girls do not uniformly express the same patterns of association between behavior problems and autonomic physiological activity. For example, Beauchaine et al. (2008) found that lower resting RSA was associated with increased aggression in males but no such relationship was observed in females. El-Sheikh and Hinnant (2011) found the initial resting RSA was negatively related to externalizing behaviors overtime for boys but not for girls. This again could be a function of a poor translation of current aggression measures to female aggressive behavior. Regardless, additional studies looking at measures of autonomic activity such as heart rate and skin conductance level similarly saw no relationship between behavior problems and physiological responding in girls (Isen et al., 2010; Fagan et al., 2017). Some posit that social and environmental influences are stronger, if not dominant, in predicting female externalizing behaviors, and that there could be sex-specific genetic effects driving the development of these behaviors (Beauchaine et al., 2008). Our correlational results support this idea as they show a direct relationship between social adversity and externalizing and internalizing behavior in females but not in males (**Table 2**).

One limitation of the current study is that we only offer two data points, 1 year apart, due to funding limitations. Although this is not sufficient to gauge a stable developmental trajectory, it does offer some preliminary examination of a developmental critical period (early childhood to pre-adolescence) and the (relative) long-term influence of physiological activity on behavior. In addition, our reports of both internalizing and externalizing behaviors were from caregivers only; having multiple informants including teacher/self-report and behavioral observation would provide a more holistic and reliable assessment. Lastly, we assessed youths from a community setting where the base rate of high scorers is generally low. Future studies integrating prospective longitudinal assessment of RSA, laboratory-based research methods, and multi-informant measures of clinical and non-clinical samples are needed to replicate our findings.

Overall, our results provide further longitudinal evidence (El-Sheikh and Whitson, 2006; Hinnant and El-Sheikh, 2009; El-Sheikh and Hinnant, 2011; Eisenberg et al., 2012) that low resting RSA may be a transdiagnostic biomarker of emotion dysregulation and that it is a predisposing risk factor for both externalizing and internalizing behavior problems during development. Further understanding of the role of RSA is consistent with the aim of the Research Domain Criteria (RDoC) initiative of the National Institute for Mental Health (Insel et al., 2010) which focuses on “new ways of classifying psychopathology based on dimensions of observable behavior and neurobiological measures” with the objective of defining “basic dimensions of functioning … cutting across disorders as traditionally defined” (Cuthbert and Insel, 2013). Our findings also highlight the importance of examining biosocial interaction effects on childhood psychopathology for males and females separately.

## Author Contributions

WZ and SF carried out data collection, data analysis and interpretation, drafting of the article, and critical revision of the article. YG carried out conception and design of the study, coordination and supervision of data collection, drafting the article, and critical revision of the article. All authors have approved the final article.

## Conflict of Interest Statement

The authors declare that the research was conducted in the absence of any commercial or financial relationships that could be construed as a potential conflict of interest.
